# Transforming and Modernizing Population and Housing Censuses in Africa: Experiences from the 2020 Round (2015-2024)

**DOI:** 10.23889/ijpds.v9i5.2727

**Published:** 2024-09-18

**Authors:** Claire Tochel, Heather Anderson, Miguel Bernabeu, Baljean Dhillon, Malihe Javidi, Tom MacGillivray, Alice McTrusty, Jon Penny, Niall Strang, Andrew Tatham, Jordan Watson, Sam Watts

**Affiliations:** 1 University of Edinburgh; 2 Princess Alexandra Eye Pavilion; 3 Royal College of Surgeons Edinburgh; 4 Glasgow Caledonian University

The objective of our organization is to assist African National Statistical Offices (NSOs) to transition from manual to digital systems, use better methods and new technologies, and increase the reliability and accessibility of their statistics.

Innovative approaches adopted to support the transformation to digital censuses include:

Use of hand-held devices at the enumeration and dissemination stages to improve data collection and usability respectively.
Use of GIS, GPS and digital remote-sensing to enable census-mapping.
Provisioning of tablets – sourcing and sharing.
Electronic operations dashboards to enable real-time monitoring of progress and quality of enumeration.
Automatic data linkage algorithms for census to post-enumeration survey matching.
Capacity building initiatives for NSO staff.

As a result, to date 29 censuses have been carried out in the 2020 Round, with another 19 planned for 2024/25. Delays have been caused by economic, security and political constraints, as well as the Covid-19 pandemic.

The implications of adopting new technologies are clear. Electronic data collection improves quality, reduces processing time and enabled enumeration during the pandemic by minimizing face-to-face contact. We will support documentation of experiences and lessons learnt in the current census round to contribute to the 2030 round.

In conclusion, the challenge of working during the pandemic gives NSOs an opportunity to ‘build back better’ and transition to full digital systems. There is a need to focus on the types of results that make the most difference e.g., on disaggregation, small area statistics, Sustainable Development Goals (SDGs) reporting, Agenda 2063 reporting, and monitoring of national development plans.

